# Global optimization of spin Hamiltonians with gain-dissipative systems

**DOI:** 10.1038/s41598-018-35416-1

**Published:** 2018-12-12

**Authors:** Kirill P. Kalinin, Natalia G. Berloff

**Affiliations:** 1 Department of Applied Mathematics and Theoretical Physics, University of Cambridge, Cambridge, CB3 0WA United Kingdom; 2 Skolkovo Institute of Science and Technology, Nobelya Ulitsa 3, Moscow, 121205 Russian Federation

## Abstract

Recently, several platforms were proposed and demonstrated a proof-of-principle for finding the global minimum of the spin Hamiltonians such as the Ising and XY models using gain-dissipative quantum and classical systems. The implementation of dynamical adjustment of the gain and coupling strengths has been established as a vital feedback mechanism for analog Hamiltonian physical systems that aim to simulate spin Hamiltonians. Based on the principle of operation of such simulators we develop a novel class of gain-dissipative algorithms for global optimisation of NP-hard problems and show its performance in comparison with the classical global optimisation algorithms. These systems can be used to study the ground state and statistical properties of spin systems and as a direct benchmark for the performance testing of the gain-dissipative physical simulators. Our theoretical and numerical estimations suggest that for large problem sizes the analog simulator when built might outperform the classical computer computations by several orders of magnitude under certain assumptions about the simulator operation.

## Introduction

Finding the global minimum of spin Hamiltonians has been instrumental in many areas of modern science. Such Hamiltonians have initially been introduced in condensed matter to study magnetic materials^1,2^ and by now they became fundamentally important in a vast spread of many other disciplines such as quantum gravity^3^, combinatorial optimization^4^, neural networks^5^, protein structures^6^, error-correcting codes^7^, X-ray crystallography^8^, diffraction imaging^9^, astronomical imaging^10^, optics^11^, microscopy^12^, biomedical applications^13^, percolation clustering^14^ and machine learning^15^.

The spin degrees of freedom in spin models are either discrete or continuous. In particular, we will be concerned with the XY model, where spins lie on a unit circle *s*_*j*_ = cos *θ*_*j*_ + i sin *θ*_*j*_, the Ising model where spins take values *s*_*j*_ = ±1 and *q*-state planar Potts model where spins take *q* discrete values. For *N* spins the classical Hamiltonians for these models can be written as

1 $$H=-\,\sum _{i=1}^{N}\sum _{j=1}^{N}\,{J}_{ij}\,\cos ({\theta }_{i}-{\theta }_{j})+\sum _{i=1}^{N}\,{g}_{i}\,\cos \,{\theta }_{i},$$

where the elements *J*_*ij*_ of matrix ***J*** define the strength of the couplings between *i*-th and *j*-th spins represented by the phases *θ*_*i*_ and *θ*_*j*_ and *g*_*i*_ is the strength of the external field acting on spin *i*. For the continuous XY model *θ*_*j*_ ∈ [0, 2*π*), for the Ising model *θ*_*j*_ ∈ {0, *π*}, and for the *q*-state planar Potts model *θ*_*j*_ = 2*πj*/*q*, *j* = 1, …, *q*.

For a general matrix of coupling strengths, ***J***, finding the global minimum of such problems is known to be strongly NP-complete^16^, meaning that an efficient way of solving them can be used to solve all problems in the complexity class NP that includes a vast number of important problems such as partitioning, travelling salesman problem, graph isomorphisms, factoring, nonlinear optimisation beyond quadratic, etc. For instance, the travelling salesman problem of a record size 85,900 has been solved by the state of the art Concorde algorithm in around 136 CPU-years^17^. The actual time required to find the solution also depends on the matrix structure. For instance, for positive definite matrices, finding the global minimum of the XY model remains NP-hard due to the non-convex constraints but can be effectively approximated using an SDP relaxation^18^ with the performance guarantee *π*/4^16^. Sparsity also plays an important role: for sufficiently sparse matrices fast methods exist^19^. As for many other hard optimisation problems, there are three types of algorithms for minimizing spin Hamiltonian problems on a classical computer: exact methods that find the optimal solution to the machine precision, approximate algorithms that generate the solution within a performance guarantee and heuristic algorithms where suitability for solving a particular problem comes from some empirical testing^20^. Exact methods can be used to solve small to medium matrix instances, as they typically involve branch-and-bound algorithms and the exponential worst-case runtime. The heuristic algorithms such as simulated annealing can quickly deliver a decent, but suboptimal (and possibly infeasible) solution^21^. Finally, global minimization of the XY and Ising models is known to be in the APX-hard class of problems^22^, so no polynomial-time approximation algorithm gives the value of the objective function that is arbitrarily close to the optimal solution (unless P = NP). The problem becomes even more challenging when the task is to find not only an approximation to the global minimum of the objective function, but also the minimisers, as needed, for instance, in image reconstruction. The values of the objective functions can be very close but for the entirely different sets of minimizers.

Recently, several platforms were proposed and demonstrated a proof-of-principle of finding the global minimum of the spin Hamiltonians such as the Ising and XY models using gain-dissipative quantum and classical systems: the injection-locked lasers^23^, the network of optical parametric oscillators^24,25^, coupled lasers^26^, polariton condensates^27^, and photon condensates^28^. In the gain-dissipative simulators, the phase of the so-called coherent centre (CC) is mapped into the “spin” of the simulator. Such CC can be a condensate^27,28^ or a coherent state generated in a laser cavity^25,26^. The underlying operational principle of such simulators depends on a gain process that is increased from below until a nonzero occupation appears via the supercritical Hopf bifurcation and the system becomes globally coherent across many CCs. The coherence occurs at the maximum occupancy for the given gain. It was suggested and experimentally verified that the maximum occupancy of the system is related to the corresponding spin Hamiltonian^27^. When the heterogeneity in densities of the CCs is removed by dynamically adjusting the gain the coherence will be established at the global state of the corresponding spin Hamiltonian^29^. We refer to these platforms as gain-dissipative analog Hamiltonian optimisers^30^ that, despite having different quantum hardware, share the basic principle that suggests the convergence to the global minimum of the spin Hamiltonian.

Here, motivated by the operation of such physical systems, we develop a new class of classical gain-dissipative algorithms for solving large-scale optimisation problems based on the Fokker-Plank-Langevin gain-dissipative equations written for a set of CCs. We show how the algorithm can be modified to cover various spin models: continuous and discrete alike. We demonstrate the robustness of such iterative algorithms and show that we can tune the parameters for the algorithm to work efficiently on various sizes and coupling structures. We show that such algorithms can outperform the standard global optimiser algorithms and have a potential to become the state of the art algorithm. Most importantly, these algorithms can be used as a benchmark for the performance of the physical gain-dissipative simulators. Finally, this framework allows us to estimate the operational time for a physical realisation of such simulators to achieve the global minimum.

The paper is organised as follows. We formulate a general classical gain-dissipative algorithm for finding the global minimum of various spin Hamiltonians in Section 1. In Sections 2 and 3 we investigate its performance on global optimisations of the XY and Ising Hamiltonians by comparing it to standard built-in global optimisers of Scipy optimisation library in Python and to the results of breakout local search and GRASP algorithms. We discuss the performance of the actual physical systems in Section 4 and conclude in Section 5.

## Gain-dissipative approach for minimising the spin Hamiltonians

The principle of operation of the gain-dissipative simulator with *N* CCs for minimisation of the spin Hamiltonians given by Eq. (1) is described by the following set of the rate equations^29,31^

2 $$\frac{d{{\rm{\Psi }}}_{i}}{dt}={{\rm{\Psi }}}_{i}({\gamma }_{i}^{{\rm{inj}}}-{\gamma }_{c}-|{{\rm{\Psi }}}_{i}{|}^{2})+\sum _{j,j\ne i}\,{{\rm{\Delta }}}_{ij}{K}_{ij}{{\rm{\Psi }}}_{j}+\sum _{q=1}^{n}\,{h}_{qi}{{\rm{\Psi }}}_{i}^{\ast (q-\mathrm{1)}}+D{\xi }_{i}(t),$$

where Ψ_*i*_(*t*) is a classical complex function that describes the state of the *i*-th CC, ${\gamma }_{i}^{{\rm{inj}}}$ is the rate at which particles are injected non-resonantly into the *i*− state, *γ*_*c*_ is the linear rate of loosing the particles, the coupling strengths are represented by Δ_*ij*_*K*_*ij*_ where we separated the effect of the particle injection that changes the strength of coupling represented by Δ_*ij*_ from the other coupling mechanisms represented by *K*_*ij*_. We consider two cases Δ_*ij*_ = 1 that physically corresponds to the site dependent dissipative coupling and ${{\rm{\Delta }}}_{ij}={\gamma }_{i}^{{\rm{inj}}}(t)+{\gamma }_{j}^{{\rm{inj}}}(t)$ appropriate for the description of the geometrically coupled condensates^29^. We also include the complex function *Dξ*_*i*_(*t*) that represents the white noise with a diffusion coefficient *D* which disappears at the threshold. The coefficients *h*_*qi*_ represent the strength of the external field with the resonance *q*:1^31^. Compared to the actual physical description^29,31^, in writing Eq. (2) we neglected the possible self-interactions within the CC and re-scaled Ψ_*i*_ so that the coefficient at the nonlinear dissipation term |Ψ_*i*_|^2^Ψ_*i*_ is 1 and allowed for several (*n*) resonant terms to be included. By writing ${{\rm{\Psi }}}_{i}=\sqrt{{\rho }_{i}}\exp [{\rm{i}}{\theta }_{i}]$ and separating real and imaginary parts in Eq. (2) we get the equations on the time evolution of the number density *ρ*_*i*_ and the phase *θ*_*i*_

3 $$\frac{1}{2}{\dot{\rho }}_{i}(t)=({\gamma }_{i}^{{\rm{inj}}}-{\gamma }_{c}-{\rho }_{i}){\rho }_{i}+\sum _{j;j\ne i}\,{{\rm{\Delta }}}_{ij}^{{\rm{inj}}}{K}_{ij}\sqrt{{\rho }_{i}{\rho }_{j}}\,\cos \,{\theta }_{ij}+\sum _{q=1}^{n}\,{h}_{qi}{\rho }_{i}^{\frac{q}{2}}\,\cos (q{\theta }_{i}),$$

4 $${\dot{\theta }}_{i}(t)=-\,\sum _{j;j\ne i}\,{{\rm{\Delta }}}_{ij}^{{\rm{inj}}}{K}_{ij}\frac{\sqrt{{\rho }_{j}}}{\sqrt{{\rho }_{i}}}\,\sin \,{\theta }_{ij}-\sum _{q=1}^{n}\,{h}_{qi}{\rho }_{i}^{\frac{q}{2}-1}\,\sin (q{\theta }_{i}),$$

where *θ*_*ij*_ = *θ*_*i*_ − *θ*_*j*_.

As we have previously shown^29,31^, the individual control of the pumping rates ${\gamma }_{i}^{{\rm{inj}}}$ is required to guarantee that the fixed points of the system coincide with minima of the spin Hamiltonian given by Eq. (1). As the injection rates ${\gamma }_{i}^{{\rm{inj}}}$ raise from zero they have to be adjusted in time to bring all CCs to condense at the same specified number density *ρ*_th_. Mathematically, this is achieved by

5 $$\frac{d{\gamma }_{i}^{{\rm{inj}}}}{dt}=\varepsilon ({\rho }_{{\rm{th}}}-{\rho }_{i}),$$

where *ε* controls the speed of the gain adjustments. If we take Δ_*ij*_ = 1 we assign *K*_*ij*_ = *J*_*ij*_. If Δ_*ij*_ depends on the injection rates, the coupling strengths will be modified, so they have to be adjusted as well to bring the required coupling *J*_*ij*_ at the fixed point by

6 $$\frac{d{K}_{ij}}{dt}=\hat{\varepsilon }({J}_{ij}-{{\rm{\Delta }}}_{ij}{K}_{ij}),$$

where $\hat{\epsilon }$ controls the rate of the coupling strengths adjustments. Equation (6) indicates that the couplings need to be reconfigured depending on the injection rate: if the coupling strength scaled by the gain at time *t* is lower (higher) than the objective coupling *J*_*ij*_, it has to be increased (decreased) at the next iteration. We shall refer to numerical realisation of Eqs (2 and 5) with Δ_*ij*_ = 1 and *K*_*ij*_ = *J*_*ij*_ and Eqs (2, 5 and 6) with ${{\rm{\Delta }}}_{ij}={\gamma }_{i}^{{\rm{inj}}}(t)+{\gamma }_{j}^{{\rm{inj}}}(t)$ and *K*_*ij*_ ≠ *J*_*ij*_ as the ‘Gain-D algorithm’ and the ‘Gain-D-mod algorithm’ respectively.

The fixed point of Eqs (3–6) are

7 $${\rho }_{i}={\rho }_{{\rm{th}}}={\gamma }_{i}^{{\rm{inj}}}-{\gamma }_{c}+\sum _{j;j\ne i}\,{J}_{ij}\,\cos \,{\theta }_{ij}+\sum _{q=1}^{n}\,{h}_{qi}{\rho }_{{\rm{t}}h}^{\frac{q}{2}-1}\,\cos (q{\theta }_{i}),$$

with the total number of particles in the system given by $M=N{\rho }_{{\rm{th}}}={\sum }_{i}\,{\gamma }_{i}^{{\rm{inj}}}-N{\gamma }_{c}$ + $\sum _{i,j;j\ne i}\,{J}_{ij}\,\cos \,{\theta }_{ij}$ +$\sum _{q}\,{\rho }_{{\rm{th}}}^{\frac{q}{2}-1}\,\sum _{i}\,{h}_{qi}\,\cos \,(q{\theta }_{i})$. Such a value of the total number of particles will be first reached at the minimum of ${\sum }_{i}\,{\gamma }_{i}^{{\rm{inj}}}$, therefore, at the minimum of the spin Hamiltonian given by

8 $${H}_{s}=-\,\sum _{i,j;j\ne i}\,{J}_{ij}\,\cos \,{\theta }_{ij}-\sum _{q}\,{\rho }_{{\rm{th}}}^{\frac{q}{2}-1}\,\sum _{i}\,{h}_{qi}\,\cos (q{\theta }_{i}\mathrm{).}$$

Eq. (8) represents the general functional that our Gain-D and Gain-D-mod algorithms optimise. By choosing which *h*_*qi*_ are non-zero we can emulate a variety of spin Hamiltonians. If *h*_*qi*_ = 0, then *H*_*s*_ represents the XY Hamiltonian. If only *h*_2*i*_ = *h*_2_ are non-zero with ${h}_{2} > {\sum }_{j;j\ne i}\,|{J}_{ij}|$ for any *i*, then the second term of the right-hand side of the Hamiltonian (8) represents the penalty, forcing phases to be 0 or *π*. It implies that the minima of *H*_*s*_ coincide with the minima of the Ising Hamiltonian. If only *h*_*qi*_ = *h*_*q*_ for *q* > 2 are non-zero, then the minima of *H*_*s*_ coincide with the minima of the *q*-state planar Potts Hamiltonian with phases restricted to discrete values *θ*_*i*_ = 2*πi*/*q*. Finally, introducing non-zero *h*_1*i*_ together with non-zero *h*_*q*_ for *q* > 1 brings the effect of an external field of strength ${g}_{i}={h}_{1i}/\sqrt{{\rho }_{{\rm{th}}}}$ in agreement with Eq. (1).

The “NP-hardness assumption” suggests that not only any classical algorithm but also any physical simulator cannot escape the exponential growth of the number of operations with the size of the problem^32^. In order to find the global minimum by evolving Eqs (2 and 5) one would require to span an exponentially growing number of various phase configurations. This can be achieved by either introducing an exponentially slow increase in the pumping rates when approaching the threshold, or by exploring an exponential growth in the number of runs using different noise seeds. In what follows we focus on the second option as it is more practical and corresponds to the operation of the actual physical simulators.

## Global minimization of the XY Hamiltonian: *h*_*qi*_ = 0

To find the global minimum of the XY Hamiltonian we numerically evolve Eqs (2 and 5) with *h*_*qi*_ = 0 using the 4th-order Runge-Kutta integration scheme.

**Figure 1:**
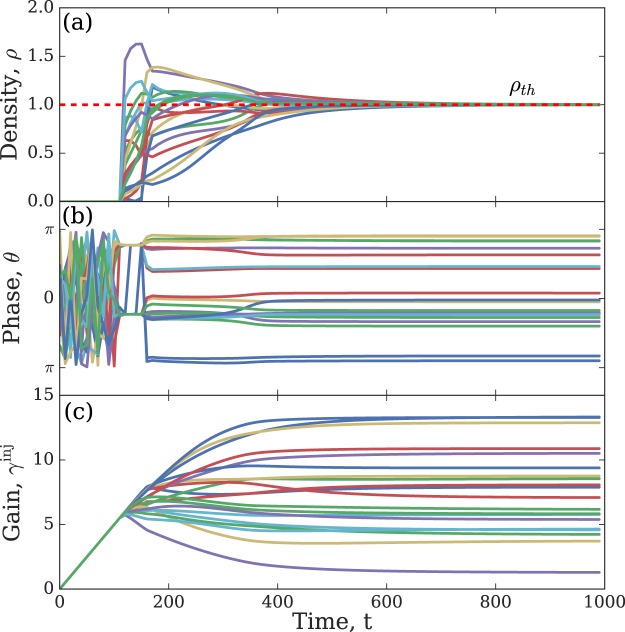
Plots of (**a**) the number densities *ρ*_*i*_ = |Ψ_*i*_|^2^ of CCs, (**b**) the phases *θ*_*i*_ and (**c**) the injection rates ${\gamma }_{i}^{{\rm{inj}}}$ as functions of time obtained by the numerical integration of Eqs (2 and 5) with *h*_*qi*_ = 0 starting with zero initial conditions for *i* = 1, …, 20.

To illustrate the operational principle of the Gain-D algorithm for minimising the XY Hamiltonian we consider *N* = 20 nodes and the coupling strengths *J*_*ij*_ that are randomly distributed between −10 and 10, see Fig. 1. Starting from a zero initial condition Ψ_*i*_ = 0, at the first stage of the evolution (while *t* < 120) the densities are well below the threshold (Fig. 1a), phases span various configurations (Fig. 1b), and all injection rates are the same (Fig. 1c). When the nodes start reaching, and in some cases overcoming the threshold, the injection rates are individually adjusted to bring all the nodes to the same value while phases stabilise to realise the minimum of the XY Hamiltonian.

A numerical approach for solving NP-hard optimisation problems depends on the scale of the problem: intermediate-scale problems can be solved with general programming tools while large-scale problems require sophisticated algorithms that exploit the structure of a particular type of objective function and are usually solved by iterative algorithms. Since the proposed Gain-D method based on Eqs (2 and 5) is an iterative algorithm, we aim to investigate its two main aspects. First, we conduct the global convergence analysis on small and mid-scale problems and verify that with the sufficient number of runs the algorithm finds a global minimum. The fact that the minimum is truly global we confirm by exploiting other optimisation methods. As for any heuristic iterative algorithm, such convergence properties can be established with confidence by performing numerous numerical experiments on different problems. Second, we perform the complexity analysis on large-scale problems with a focus on how fast the algorithm converges per run.

To characterise the performance of the Gain-D algorithm, we compared it to the heuristic global optimisation solvers such as direct Monte Carlo sampling (MC) and the basin-hopping (BH) algorithm. Both methods are built-in optimisation algorithms of a well-known Scipy optimisation library in Python so their performance has been carefully tested. The BH algorithm depends on a local minimisation algorithm performing the optimal decent to a local minimum at each iteration. We considered several local minimisation methods as applied to the minimisation of the XY Hamiltonians and determined that the quasi-Newton method of Broyden, Fletcher, Goldfarb, and Shanno (L-BFGS-B)^33,34^ has shown the best performance (see Supplementary Information). The L-BFGS-B algorithm is a local minimisation solver which is designed for large-scale problems and shows a good performance even for non-smooth optimisation problems^33,34^. At each run of the MC algorithm, we generate a random starting point and use L-BFGS-B algorithm to find the nearest local minimum. These minima are compared to find the global minimum. Applying simple Monte-Carlo method to the random coupling matrices allows one to understand how hard these instances are, while the hardness or the easiness of the problems is a critical issue to address for understanding the performance of a newly suggested algorithm, i.e. Gain-D algorithm. The BH algorithm is a global minimisation method that has been shown to be extremely efficient for a wide variety of problems in physics and chemistry^35^ and to give a better performance on the spin Hamiltonian optimisation problems than other heuristic methods such as simulated annealing^36^. It is an iterative stochastic algorithm that at each iteration uses a random perturbation of the coordinates with a local minimisation followed by the acceptance test of new coordinates based on the Metropolis criterion. Again L-BFGS-B algorithm has shown the best performance as a local optimiser at each step of the BH algorithm. Both BH and MC algorithms were supplied with the analytical Jacobian of the objective function for better performance results.

**Figure 2:**
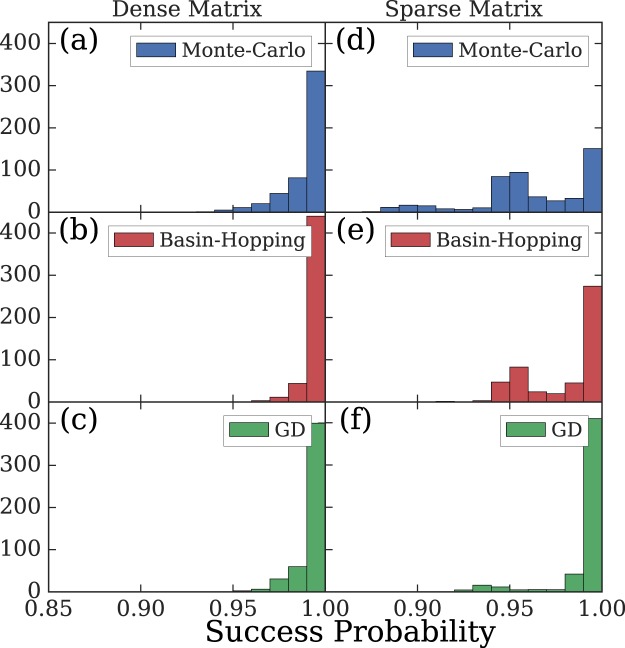
The success probability of (**a**,**d**) MC, (**b**,**e**) BH, and (**c**,**f**) Gain-D algorithms when minimising the XY Hamiltonian for the matrix size *N* = 50. The results of 500 runs are averaged over 50 real symmetric coupling matrices *J* with the elements randomly distributed in [−10, 10] for (**a**–**c**) ‘dense’ and (**d**–**f**) ‘sparse’ matrices described in the main text. The number of internal BH iterations was set to ten to bring about a similar performance to the Gain-D algorithm for ‘dense’ matrices.

To confirm the global convergence, we compared the Gain-D algorithms to the BH and MC algorithms by minimizing XY Hamiltonian for various matrices. The numerical parameters and the initial conditions for the Gain-D algorithm described by Eqs (2 and 5). In particular, we generated 50 real symmetric coupling matrices **J** = {*J*_*ij*_} of two types. We considered ‘dense’ matrices with elements that are randomly distributed in [−10, 10] and ‘sparse’ matrices where each CC is randomly connected to exactly three other CCs with the coupling strengths randomly generated from the interval with the bounds that are randomly taken from {−10, −3, 3, 10}. For each such matrix, we ran the Gain-D, BH and MC algorithms starting with 500 random initial conditions for the BH and MC algorithms and with zero initial conditions and 500 different noise seeds for the Gain-D algorithm. The values of the global minimum of the objective function found by the Gain-D algorithm and the comparison methods were found to match to ten significant digits. For ‘dense’ matrices the success probabilities of the Gain-D algorithms were similar to both comparison methods. The distribution of success probabilities over various ‘dense’ matrix instances is shown in Fig. 2(a–c) for *N* = 50 and suggests that for such matrices the systems have very narrow spectral gap so the distributions are densely packed for probabilities over 93% for the MC, 96% for the BH, and 95% for the Gain-D algorithm. It is known that the spectrum of random matrices is simple^37^, so more difficult instances can be specifically constructed as illustrated in Fig. 2(d–f), where the Gain-D algorithm greatly outperforms the comparison algorithms on ‘sparse’ matrices. Thus, we established the global convergence properties of the proposed Gain-D algorithms on various problems and verified that the Gain-D algorithms finds the global minimum. The further advantages of the Gain-D algorithms over the best classical optimisers for some particular types of the coupling matrices are elucidated elsewhere^38^.

## Global minimization of the Ising Hamiltonian: *h*_2*i*_ = *h*_2_ ≠ 0

**Figure 3:**
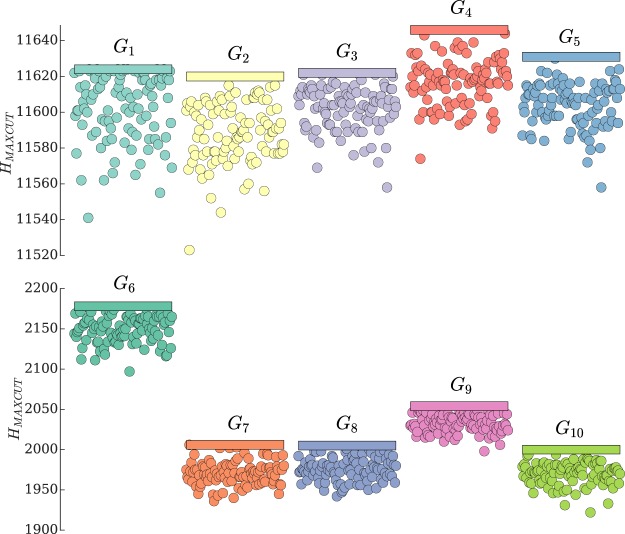
The performance of the Gain-D algorithm (2–5) for solving the MaxCut optimisation problem on G-Sets {*G*_1_–*G*_10_} of size *N* = 800. The known optimal values are plotted with coloured rectangles for each *G*_*i*_. The results of the Gain-D algorithm are shown with scatters for 100 runs on each *G*_*i*_. The average MaxCut values are within 0.2–0.3% (1.1–1.8%) of the optimal solution for *G*_1_–*G*_5_ (*G*_6_–*G*_10_) sets. The time per each run of the Gain-D algorithm has been fixed to around 35–40*s* for all G-Sets.

To find the global minimum of the Ising Hamiltonian we solve Eqs (2 and 5) with *h*_*qi*_ = 0 if *q* ≠ 2 and *h*_*qi*_ = *h*_2_ numerically. Based on these equations we test the Gain-D algorithm by finding the maxima of MaxCut optimisation problem on the well-known G-Set instances^39^ and summarise our findings in Fig. 3. The optimal MaxCut values^40^ are plotted with coloured rectangles and the solutions of the Gain-D algorithm are shown with scatters for 100 runs for each G instance. The algorithm demonstrates good performance with the average found cuts being within 0.2–0.3% for *G*_1_–*G*_5_ and 1.1–1.8% for *G*_6_–*G*_10_ of the optimal solutions. The same numerical parameters were used for all simulations. The computational time for finding each cut has been limited by the same value of 35–40s, single core simulations on MacBook Pro, 2.7 GHz Intel Core i7, 16 GB 2133 MHz LPDDR3, 2.7 GHz Intel Core i7, 16 GB 2133 MHz LPDDR3. The time performance of the state of the art algorithms depends on a particular problem and for *G*_1_–*G*_10_ varies from 13*s* to 317*s* (A C++ implementation of BLS algorithm was run on an Intel Xeon E5440 with 2.83 GHz and 2 GB in^40^) and is within 100–854*s* for GRASP tabu search^41^ algorithms are programmed in C and compiled using GNU though their solutions are much less deviated from the optimal values. We also confirmed that for the coupling matrix of size N = 10000, namely, G70 problem from the G-Set, all found solutions of the MaxCut problem out of 100 runs, 1000 time iterations are within 1.1% of the global minimum from the known optimal solution^40^. These results are achieved with an average computational time per run of 530*s* compared to 11365*s* of the BLS algorithm. Therefore, the proposed Gain-D algorithm is highly competitive with the existing state of the art MaxCut algorithms at least regarding the computational time. The deviation of solutions from the optimal values can be further reduced by tuning the parameters *ρ*_th_ and *ε* or by investigating the extensions to the Gain-D algorithm. Among such possible modifications is the introduction of individual dynamic rates of the gain adjustments *ε*_*i*_(*t*).

## Projected performance of the Gain-D simulators

So far we discussed the implementation of the Gain-D algorithms on a classical computer. An actual physical implementation of these algorithms on simulators will enjoy a super-fast operation and parallelism in processing various phase configurations as the system approaches the global minimum from below even if the system behaves entirely classically. Further acceleration could be expected if quantum fluctuations and quantum superpositions contribute to scanning the phase configurations. The times involved into the hardware operation of the Gain-D simulators vary on the scale of pico- to milli-seconds. For instance, in the system of non-degenerate optical parametric oscillators (NOPO) the time-devision multiplexing method is used to connect a large number of nodes and the couplings are realised by mutually injecting with optical delay lines with the cavity round trip time being of the order of *μ*s^25^, it takes an order of 100 picoseconds for the polariton graphs to condense^27^ and 10 ps to 1 ns for photon condensates^28^. The feedback mechanism can be implemented via optical delay lines (in NOPO system), by holographic reconfiguration of the injection via the spatial light modulator or mirror light masks (e.g. by DLP high-speed spatial light modulators) in solid-state condensates or by electrical injection (e.g. in the polariton lattices^42^).

**Figure 4:**
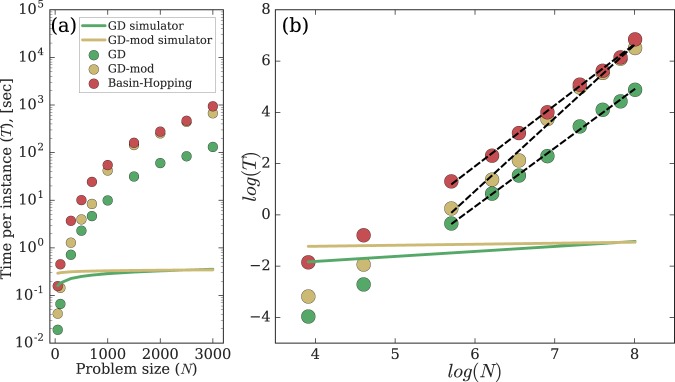
The performance of the Gain-D, Gain-D-mod, and BH algorithms in minimizing the XY Hamiltonian with *N* up to 3000. (**a**) The time per instance *T* as a function of the problem size *N*. In the case of Gain-D and Gain-D-mod algorithms, *T* is the time averaged over 20 runs necessary to reach a stationary state. For the BH algorithm, this time consists of ten internal BH iterations necessary to have about the same success probabilities as the Gain-D algorithm. (**b**) *T* as a function of *N* in the logarithmic scale. The performance of the algorithms are fitted by the linear interpolation functions 2.29 *log* *N* − 13.42, 2.85 *log* *N* − 16.2, and 2.38 *log* *N* − 12.39, for the Gain-D, Gain-D-mod, and BH algorithms, respectively. The projected performance of the Gain-D simulator dominated by the dissipative (gain) coupling is shown with solid green (yellow) lines whose linear asymptotic in (**b**) is 0.2 *log* *N* − 2.6 (0.04 *log* *N* − 1.38).

The number of runs one needs to reliably find the global minimum grows with the size of the problem *N*. This growth is expected to be exponential for any algorithm (if *P* ≠ *NP*). However, we can compare how time per run grows with the problem size for considered algorithms. We perform the complexity analysis on mid- and large-scale problems and summarise the results in Fig. 4. The Gain-D algorithm demonstrates the consistent speedup over the BH algorithm for all problem sizes *N* in Fig. 4(a). The log plot in Fig. 4(b) indicates that both algorithms show polynomial time per run with the complexity of the Gain-D algorithm being close to *O*(*N*^2.29^). Note that such polynomial scaling does not preclude the exponential growth of the algorithm due to the “NP-hardness assumption”^32^.

Next we estimate the time scale on which the analog physical simulators can be expected to operate. For instance, the polariton (photon) lattices span the phases on a pico-second scale which can be neglected in comparison with the feedback and adjustment times. By taking an upper limit on this feedback time as 0.1 *ms*^43^ and counting the number of such adjustments in the Gain-D and Gain-D-mod algorithms, i.e. the average number of time iterations required to reach a fixed point per run, we can estimate the upper bound of the time needed by the physical implementation of the Gain-D simulator to find the global minimum. Since the physical simulators benefit from the built-in parallelism in choosing among various phase configurations as the system is pumped from below, we assume that the time it takes to bring the system to the threshold is independent of the number of nodes *N*. Under these assumptions, the estimated time-performance of the real physical simulators in minimising the Ising or XY Hamiltonians is shown in Fig. 4 by the solid green (yellow) lines for the Gain-D (Gain-D-mod) simulators. For large *N* from Fig. 4 we estimate the speed-up of the Gain-D simulators in comparison with the classical computations to be of the order of 10^−5^*N*^2^–10^−7^*N*^3^. Due to the adaptive setting of the coupling matrix in the Gain-D-mod algorithm, the number of time iterations grows slower with the size of problem *N* than for the Gain-D algorithm so that the performance of the Gain-D simulator can possibly be surpassed by the Gain-D-mod simulator for large *N*.

## Conclusions

Motivated by a recent emergence of a new type of analog Hamiltonian optimisers – the gain-dissipative simulators – we formulate a novel gain-dissipative algorithm for solving large-scale optimisation problems which is easily parallelisable and can be efficiently simulated on classical computers. We show its computational advantages in comparison with built-in methods of Python’s Scipy optimisation library in minimising XY Hamiltonian and the state-of-the-art methods in solving MaxCut problem. We argue that the generalisation of the Gain-D algorithm for solving different classes of *NP*-hard problems can be done for both continuous and discrete problems and demonstrate it by solving quadratic continuous and binary optimisation problems. The Gain-D algorithm has a potential of becoming a new optimisation algorithm superior to other global optimisers. This algorithm allows us to formulate the requirement for the simulators hardware built using a system of gain-dissipative oscillators of different nature. Our algorithm, therefore, can be used to benchmark the existing gain-dissipative simulators. When the run-time of the classical algorithm is interpreted in terms of the time of the actual operation of the physical system one might expect such simulators to greatly outperform the classical computer.

Finally, we would like to comment on classical vs quantum operation of such simulators. When a condensate (a coherent state) is formed – the system behaves classically as many bosons are in the same single-particle mode and non-commutativity of the field operators can be neglected. However, the condensation process by which the global minimum of the spin models is found involves quantum effects. It was shown before, that the condensation process can be described by a fully classical evolution of the Nonlinear Schrödinger equation that takes into account only stimulated scattering effects and neglects spontaneous scattering^44^. The classical or quantum assignment to gain-dissipative simulators depends on whether quantum fluctuations and spontaneous scattering effects during the condensation provide a speed-up in comparison with entirely classical noise and stimulated scattering. This is an important question to address in the future research on such simulators and the comparison with the classical algorithm that we developed based on the gain-dissipative simulators architecture allows one to see if the time to find the solution scales better than with the best classical algorithms.

## Electronic supplementary material

Supplementary Information
