# Supplementary material for: Global optimization of spin Hamiltonians with gain-dissipative systems
Source: Sci Rep. 2018 Dec 12;8:17791. doi: 10.1038/s41598-018-35416-1 (PMC6290793; doi:10.1038/s41598-018-35416-1)
Supplement: Supplementary file 1 — Supplementary Information [file 41598_2018_35416_MOESM1_ESM.pdf]

Supplementary Information: Global optimization of spin Hamiltonians with gain-dissipative systems

Kirill P. Kalinin[1] and Natalia G. Berloff [2,1]
[1]Department of Applied Mathematics and Theoretical Physics, University of Cambridge, Cambridge CB3 0WA, United Kingdom
[2]Skolkovo Institute of Science and Technology Novaya St., 100, Skolkovo 143025, Russian Federation

Performance of local optimisation algorithms for the global minimization of the XY Hamiltonian

At each iteration of the Monte-Carlo and the Basin-Hopping methods we use the L-BFGS-B algorithm, since it has shown better results for the global minimization of the XY Hamiltonian regarding both performance and the quality of solution compared to other available algorithms in scipy.optimize.minimize library such as the sequential least squares programming (SLSQP), nonlinear conjugate gradient algorithm (CG), truncated Newton (TNC) algorithm, and BFGS. In comparison with the BFGS algorithm, the L-BFGS-B (limited memory BFGS) algorithm exploits an estimation of the inverse Hessian matrix. Each algorithm was supplied with the analytical Jacobian. The performance of the algorithms is shown in Fig. S1. The L-BFGS-B algorithms is the fastest in comparison with all the other algorithms (see Fig. S1(a,b)) while the success probabilities are comparable (Fig. S1(c)).

The numerical parameters and the initial conditions for the Gain-D algorithm described by Eqs (2 and 5) are as follows:

$$\epsilon = \epsilon_0 \max_i \sum_j |J_{ij}|,$$

$$\rho_{th} = \rho_{0,th} \max_i \sum_j |J_{ij}|,$$

$$\gamma_i^{inj}(t=0) - \gamma_C = -\max_i \sum_j |J_{ij}|,$$

$$\rho_i(t=0) = \theta_i(t=0) = 0.$$

For Gain-D-mod algorithm Eqs. (2, 5 and 6) we choose $\hat{\epsilon} = 1$ in addition to the parameters listed above. For the XY problem $\epsilon_0 = 0.05$ and $\rho_{0,th} = 0.1$, for the MaxCut $\epsilon_0 = 0.005$ and $\rho_{0,th} = 0.15$. For the MaxCut problems an external

resonant field was slowly activated with $h_2 = 0.05(\tanh(3t/t_{max} - 1.5) + 1) \times \max_i \sum_j |J_{ij}|$.

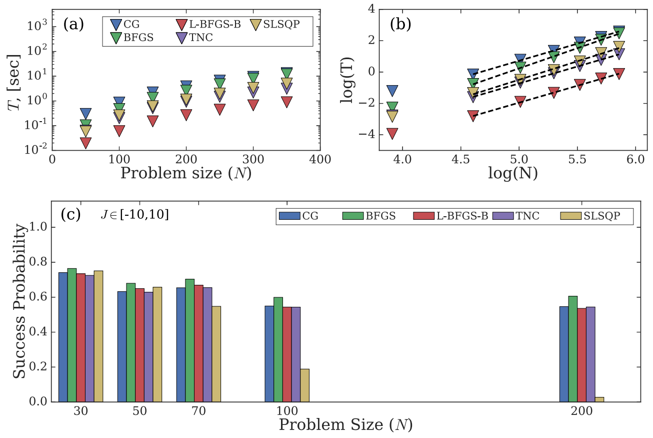

FIG. S1: The performance of the various local optimisers when finding the global minimum of the XY Hamiltonians for matrix sizes up to $N = 200$. The run-time dependence on the matrix size N is shown in (a) and in a log scale in (b). The success probability of 99% is shown in (c) where each algorithm starts from the same 100 random initial states. The probabilities are averaged over 25 dense coupling matrices with randomly generated elements in $[-10, 10]$.
